# Partial Characterization of an Enzymatic Extract from Bentong Ginger (*Zingiber officinale* var. Bentong)

**DOI:** 10.3390/molecules190812336

**Published:** 2014-08-15

**Authors:** Ahmad Nafi’, Foo Hooi Ling, Jamilah Bakar, Hasanah M. Ghazali

**Affiliations:** 1 Faculty of Food Science and Technology, Universiti Putra Malaysia, 43400 UPM Serdang, Selangor DE, Malaysia; E-Mails: ama_nafi@yahoo.com (A.N.); jamilah@upm.edu.my (J.B.); 2 Faculty of Agricultural Technology, University of Jember, of Jl Kalimantan 37 Tegalboto Jember 68121 East Java, Indonesia; 3 Faculty of Biotechnology and Biomolecular Sciences, Universiti Putra Malaysia, 43400 UPM Serdang, Selangor Darul Ehsan, Malaysia; E-Mail: hlfoo@upm.edu.my

**Keywords:** stabilizer, biochemical properties, storage stability

## Abstract

Extraction of protease from a local ginger rhizome (*Zingiber officinale* var. Bentong) was carried out. The effect of extraction pH (6.4, 6.8, 7.0, 7.2, 7.6, 8.0, 8.4, and 8.8) and stabilizers (0.2% ascorbic acid, 0.2% ascorbic acid and 5 mM EDTA, or 10 mM cysteine and 5 mM EDTA) on protease activity during extraction was examined. pH 7.0 potassium phosphate buffer and 10 mM cysteine in combination with 5 mM EDTA as stabilizer were found to be the most effective conditions. The extraction procedure yielded 0.73% of Bentong ginger protease (BGP) with a specific activity of 24.8 ± 0.2 U/mg protein. Inhibitory tests with some protease inhibitors classified the enzyme as a cysteine protease. The protease showed optimum activity at 60 °C and pH 6–8, respectively. The enzyme was completely inhibited by heavy metal cations such as Cu^2+^, and Hg^2+^. SDS stimulated the activity of enzyme, while emulsifiers (Tween 80 and Tween 20) slightly reduced its activity. The kinetic analysis showed that the protease has *K_m_* and V_max_ values of 0.21 mg mL^−1^ and 34.48 mg mL^−1^ min^−1^, respectively. The dried enzyme retained its activity for 22 months when stored at −20 °C.

## 1. Introduction

Ginger, known botanically as *Zingiber* *officinale* Roscoe, has its origin in South Asia but has spread to many other regions of the world, where it is is used in foods or other applications. In ancient times, ginger was used as medicine in India, China and Europe. Today, ginger is one of the most important and widely used spices in the world [1]. Ginger is also used in pharmacy due to the presence in the rhizome of the phenolic substances gingerol and shagaol [2] which are reported to have anti-cancer [3] and antioxidant activities [2]. Since 1973, ginger was identified as one of the sources of protease with an irrefutable proteolytic activity. Currently, the sale of protease constitutes the largest enzyme trade in the global market and is forecasted to reach $ 3.74 billion in 2015 [4]. In order to fulfill market needs, researchers have explored proteases from animal [5], plant [6] and microbial [7] sources. Among the plant proteases, bromelain from pineapple (*Ananas comosus*) and papain from papaya (*Carica papaya*) are the main industrial proteases, but they account for only 8% of the market share [8]. Hence, researchers keep searching for new sources of plant protease to meet industrial needs.

Ginger protease (GP) or zingibain, which was first reported as a new source of protease in 1973, exhibits remarkable proteolytic activity [9]. GP is very active meat tenderizer against collagen and other connective tissue proteins [9,10,11,12]. Good milk clotting activity is also attributed to GP [6] and, therefore, it is used in the preparation of ginger milk curd in the south of China.

A previous study regarding characterization of protease extracted from common ginger (*Zingiber* *officinale* Roscoe) showed an optimum activity at 60 °C with a broad pH range of 6 to 8. The enzyme, is considered as a cysteine protease due to its inhibition by NEM and Hg^2+^ and Cu^2+^ and the fact it retains its activity in the presence of monocations except Li^+^ and some detergents [13]. The protease in ginger rhizome could be extracted using buffer or organic solvents such as ethanol and acetone. Sodium phosphate buffer pH 7 and Tris-HCl buffer pH 8 have been used to extract the protease [8,12]. Cysteine enhanced the thermostability of a ginger protease while ethylenediamine tetraacetic acid (EDTA) was not an effective stabilizer [8]. Ascorbic acid was also reported to prevent oxidation from occurring during GP extraction hence improving its stability [6,8]. In order to enhance the stability of GP, a combination of those stabilizers needs to be studied.

Malaysia, one of the ginger-producing countries in Southeast Asia, produced 9.017 metric tons of ginger rhizomes in 2012 [14]. One of the exotic ginger varieties grown in Malaysia is the Bentong ginger or *halia* Bentong (*Zingiber* *officinale* Var. Bentong) [15]. This variety is cultivated only at Bentong, a highland in Malaysia. Bentong ginger shows a different phenolic substances profile [16] and anti-oxidative activity [17] in comparison with other Malaysian gingers. In order to develop a possible value-added usage for this ginger in comparison with common ginger, a study was conducted to examine the properties of BGP following extraction and partial purification.

## 2. Results and Discussion

### 2.1. Extraction of Ginger Protease (BGP) and Effect of Stabilizers

**Figure 1:**
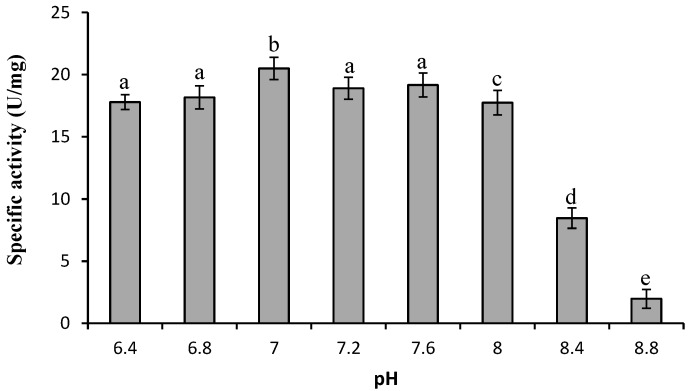
Effect of extraction pH on activity of Bentong ginger protease. Different letters indicate significant differences at *p* ≤ 0.05.

The pH and the presence or absence of a stabilizer played a significant role during the extraction of protease from ginger rhizomes. The activity of the enzyme was significantly higher (*p* ≤ 0.05) when extracted at neutral pH (Figure 1) compared to acidic or alkaline pH. This result is similar to that of a previous study which successfully produced protease from ginger using phosphate buffer at pH 7 [12,13].

The activity of BGP during extraction was stabilized greatly by addition of EDTA (Table 1) and 10 mM cysteine. Ascorbic acid (0.2% w/v) in combination with EDTA showed higher stabilization effect than ascorbic acid alone for a duration of up to 4 days.

**Table 1:** Effect of extraction stabilizers on activity of Bentong ginger protease.Means in the same row with the same superscript capital letter are not significantly different (*p* ≤ 0.05). Means in the same column with the same superscript small letter are not significantly different (*p* ≤ 0.05).

Day of Storage	Enzyme Activity (unit/mL) of Different Stabilizers at 4 °C			
	Control	0.2% Ascorbic Acid	0.2% Ascorbic Acid + 5 mM EDTA	10 mM Cysteine + 5 mM EDTA
0	11.7 ± 0.2 ^Aa^	25.9 ± 0.4 ^Ba^	30.7 ± 0.5 ^Ca^	32.6 ± 0.4 ^Ca^
1	5.0 ± 0.2 ^Ab^	23.8 ± 0.2 ^Ba^	29.0 ± 0.1 ^Cb^	31.9 ± 0.5 ^Da^
2	1.6 ± 0.2 ^Ac^	20.8 ± 0.4 ^Bb^	28.8 ± 0.3 ^Cb^	31.4 ± 0.2 ^Db^
3	0.7 ± 0.2 ^Ac^	19.4 ± 0.3 ^Bc^	28.4 ± 0.3 ^Cb^	30.9 ± 0.4 ^Db^
4	0.3 ± 0.1 ^Ac^	17.4 ± 0.3 ^Bd^	27.1 ± 0.5 ^Cc^	29.3 ± 0.2 ^Dc^

**Figure 2:**
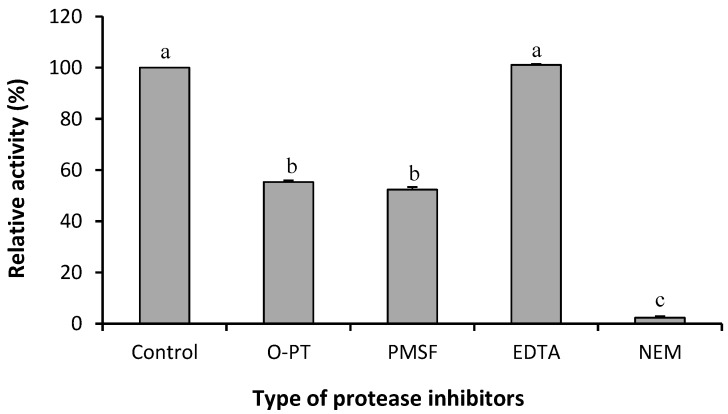
Effect of inhibitors on the activity of Bentong ginger protease. *O*-PT: *o*-phenanthroline; PMSF: Phenylmethylsulfonyl fluoride; EDTA: Ethylenediamine tetra-acetic acid; NEM: *N*-ethylmaleimide. Different superscripts indicate significant difference (*p* ≤ 0.05).

This result is similar to that of a previous study which successfully produced protease from common ginger [13]. Cysteine is an anti-autolysis agent effective for protecting the stability of protease during extraction [8]. EDTA improved the stability of the protease activity of both crude extract (Figure 2) and dried BGP. In solution, EDTA chelates the metal ions that could lead to inactivation of the enzyme by attacking the sulfhydryl group in the locus of cysteine protease. EDTA also could protect the enzyme from oxidation occurring during extraction [18]. Without any stabilizers, the activity of crude ginger proteases decreased drastically during extraction and storage.

The method used for the production of lyophilized BGP yielded 0.73% (w/w of fresh ginger rhizome) of dry BGP with a specific activity of 24.8 ± 0.2 Unit/mg protein. The current method used in this study is comparable with those employed in the previous studies. A protease extraction using acetone followed by 60% (w/v) ammonium sulfate precipitation to extract and concentrate ginger protease from China obtained a yield of 0.6% (w/v) protease [19]. However, BGP’s yield and specific activity was lower than that of common ginger protease which has yield of 0.94% (w/w of fresh weight) with a specific activity of 27.6 ± 0.1 Unit/mg [16]. The different varieties of ginger rhizome (common and Bentong) used probably caused that differences in yield and specific activity.

### 2.2. Characterization of Ginger Protease

#### 2.2.1. Determination of Protease Type

The activity of the BGP under study was found to be greatly inhibited by NEM (Figure 2), therefore, this protease is most likely a cysteine protease. By using leupeptin, iodoacetic acid and E-64 (*trans*-l-epoxysuccinylleucylamido-(4-guanidino)-butane), Su *et al.* [6] also concluded that ginger protease was a cysteine protease. However, the ginger protease was significantly inhibited by PMSF and *o*-phenanthroline (Figure 2). This phenomenon is similar to cysteine protease extracted from common ginger [13] and garlic, whose activity was also inhibited by PMSF [20]. Furthermore, PMSF and *o*-phenanthroline also reduced the activity of a cysteine extracted from capsules of caper (*Capparis* *spinosa*) [21]. The reduction of protease activity was most likely to be attributed to the occurrence of PMSF bound to serine and *o*-phenanthroline complexed with alanine, phenylalanine or tryptophan residues on the non-active side of BGP, thereby reducing the affinity of the substrate with the enzyme and subsequently reducing the enzyme activity. In addition, Frey and Hegeman [22] reported that no reagents are exactly specific to a certain nucleophilic group, but also can react with other groups.

#### 2.2.2. Optimum Temperature

**Figure 3:**
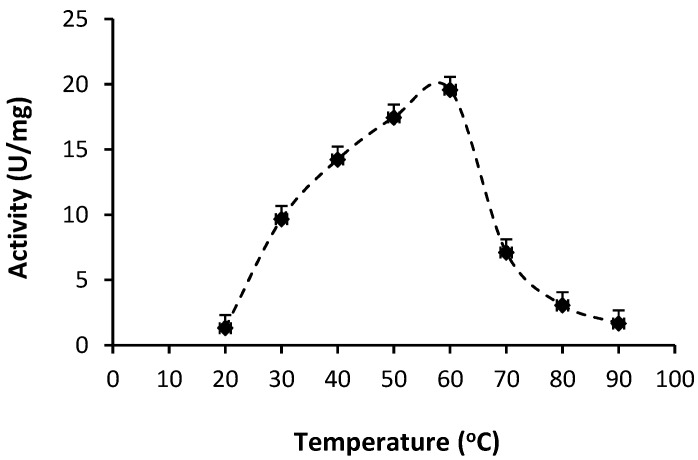
Effect of temperature on activity of Bentong ginger protease.

The optimum temperature of BGP was 60 °C (Figure 3). The result obtained is similar to the findings of [13,20] who reported that ginger protease exhibited broad optimal proteolytic activity from 40 to 60 °C and lost its activity when the temperature increased to 70 °C. The loss in activity at 70 °C was 64% in the present study due to denaturation of the enzyme. With its high optimum temperature, BGP shows potential for food industry applications such as milk coagulation [23] and as a meat tenderizing agent [11].

#### 2.2.3. Optimum pH

**Figure 4:**
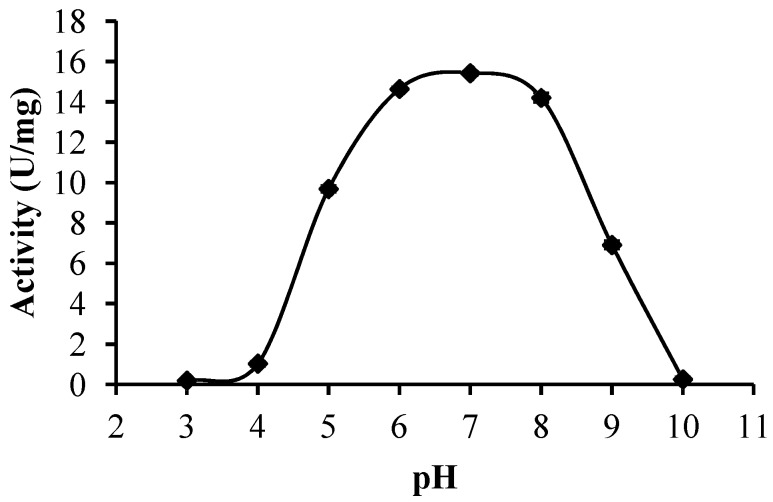
Effect of pH on activity of Bentong ginger protease.

BGP was found to have an optimum pH that ranged from 6.0 to 8.0 (Figure 4). A similar result was obtained in a previous study [13]. Other plant proteases, such as procerain, a protease extracted from *Callotropis procera*, showed an optimal hydrolysis of azoalbumin at pH values ranging from 7.0 to 9.0 [24].

A protease extracted from kesinai (*Streblus asper*) leaves showed optimum activity on pH 7.5 [25]. The protease of crude preparation was detected in this research displayed a broad optimum pH range, hence warranting broad application in various food processing uses.

#### 2.2.4. Effect of Cations and Detergents

The thiol-blocking heavy metal cations of Cu^2+^ and Hg^2+^ were found to significantly inhibit the proteolytic activity of BGP (*p* ≤ 0.05, Table 2). In comparison, monovalent cations have no significant effect on BGP. However, Li^+^ and divalent cations such as Co^2+^ and Zn^2+^ demonstrate moderate inhibition of 50.6%, 59.5% and 33.5%, respectively. The reduction in BGP protease activity was most likely due to the sulfhydryl group in the active site of BGP being affected by those metal cations and causing the inactivation of cysteine protease. A similar result was reported by Demir *et al.* [21], where metal cations such as Hg^2+^ and Cu^2+^ caused substantial inhibition of a cysteine protease, capparin, extracted from capsules of caper (*Capparis spinosa*). These result also showed agreement with previous study on effect of cation on proteolytic activity of common ginger protease [13].

**Table 2:** Effect of cations on the activity of Bentong ginger protease.The same letterindicates not significantly different at *p* ≤ 0.05.

Cations	Relative Activity (%)
Control	100.0 ± 0.0 ^a^
K^+^	96.3 ± 3.4 ^a^
Na^+^	100.7 ± 1.1 ^a^
Li^+^	50.6 ± 3.2 ^b^
Mg^2+^	89.0 ± 1.3 ^c^
Ba^2+^	79.9 ± 2.2 ^d^
Ca^2+^	76.4 ± 0.6 ^e^
Co^2+^	59.5 ± 2.7 ^f^
Zn^2+^	33.5 ± 2.2 ^g^
Cu^2+^	4.7 ± 2.6 ^h^
Hg^2+^	0.3 ± 0.1 ^i^
Al^3+^	92.6 ± 3.4 ^c^

**Figure 5:**
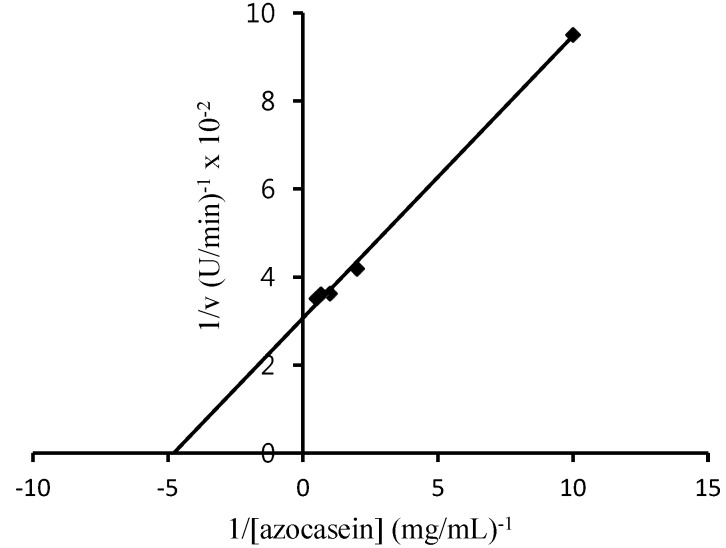
Lineweaver-Burk plot for the effect of substrate concentration on activity of the ginger protease.

**Figure 6:**
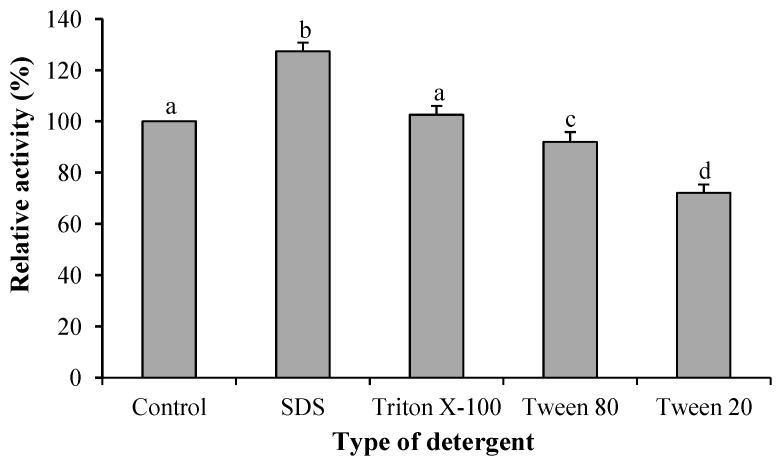
Effect of detergents on activity of ginger protease. Different superscripts indicate a significant difference (*p* ≤ 0.05).

The effects of detergents tested in this experiment are shown in Figure 5. Interestingly, SDS at 0.25 mM enhanced the activity of BGP by 27.4%. The effect of detergents on proteolytic activity of BGP was similar that seen with common ginger protease [13]. A similar result was also reported by Demir *et al.* [21], where 0.1 mM SDS stimulated the activity of capparin by 47%, but a higher concentration of SDS at either 1 or 10 mM would completely inhibit its proteolytic activity. SDS at low concentrations could mask the intrinsic charge of protein [26]. Being negatively charged, SDS tends to attract the hydrogen atom of the sulfhydryl group of cysteine. The ionized cysteine at the active site of BGP possibly facilitates the binding reaction of substrate-enzyme and, therefore, contributes to the increment in the activity of BGP by 27%. Triton X-100 as a nonionic detergent has no inhibitory effect while an emulsifier detergent, such as Tween 20 and Tween 80, significantly reduced (*p* ≤ 0.05) the proteolytic activity of BGP (Figure 6). The negative effect of these emulsifiers most probably caused the change of the enzyme’s conformation when the hydrophobic amino acid residues were disturbed by the nonpolar sites of the emulsifiers.

#### 2.2.5. *K_m_* and V_max _ Value of BGP

The calculated *K_m_* and V_max _ values of BGP were 0.21 mg mL^−1^ and 34.48 mg mL^−1^ min^−1^, respectively. Kinetics analysis of common ginger protease showed that it has lower *K_m_* and higher V_max _ values of 0.18 mg mL^−1^ and V_max_ of 35.74 mg mL^−1^ min^−1^ (figure not shown). The low *K_m_* value of BGP indicated that the enzyme could easily react with the substrate with a high rate of hydrolysis.

#### 2.2.6. Storage Stability of BGP

The dried BGP showed high stability at low temperature (−20 °C), but 46% of the activity was lost after 90 days at 4 ± 1 °C and drastically lost its proteolytic activity when stored at room temperature (Table 3). The half-life, t_1/2_, of BGP increased from 3 days at room temperature to 3.5 months at 4 ± 1 °C and 22 months at −20 °C. The t_1/2 _ of this enzyme kept at −20 °C was higher than that of ginger protease extracted using acetone precipitation which has a t_1/2_ of 18 months [8]. However it lower than that of common ginger protease which has t_1/2_ of 24.5 months (data not shown).

**Table 3:** Effect of storage temperature on activity of bentong ginger protease.

Time (day)	Relative Activity (%) at Different Storage Temperature		
	RT *	4 ± 1 °C	−20 ± °C
0	100.0 ± 0.0	100.0 ± 0.0	100.0 ± 0.0
1	67.7 ± 1.1	87.1 ± 0.4	99.3 ± 0.1
2	47.1 ± 1.1	85.3 ± 0.2	98.5 ± 0.1
3	52.7 ± 0.7	83.2 ± 0.1	98.5 ± 0.1
4	37.0 ± 1.1	81.8 ± 0.7	98.1 ± 0.3
5	22.7 ± 2.3	78.8 ± 0.2	97.6 ± 0.3
10	16.3 ± 2.1	76.6 ± 0.3	96.9 ± 0.3
20	12.9 ± 2.3	74.0 ± 0.7	95.2 ± 0.1
30	9.9 ± 2.3	66.6 ± 0.2	94.4 ± 0.4
60	8.2 ± 3.3	62.1 ± 0.3	93.2 ± 0.4
90	3.4 ± 1.9	56.1 ± 0.8	93.6 ± 0.2

* RT (room temperature).

## 3. Experimental Section

### 3.1. Materials and Methods

Ginger rhizomes (Bentong variety) of commercial maturity were purchased from wet markets in Sri Kembangan, Malaysia. The ginger plants were grown in a highland area located about 700 meters above sea level. Three different batches of the ginger were used. Azocasein, cysteine, ethylenediamine tetra acetic acid (EDTA) and ascorbic acid were purchased from Sigma-Aldrich, (St. Louis, MO, USA). Other chemicals used in this experiment were of analytical grade.

### 3.2. Extraction of Bentong Ginger Protease (BGP)

The ginger rhizomes were first cleaned under running tap water and dried in the sun for 24 h prior to extraction. They were then finely chopped and 100 g were homogenized with 200 mL of 100 mM pottasium phosphate buffer using a Waring blender (New Hartford, CT, USA). The homogenate was then filtered through a piece of cheesecloth following which the filtrate was centrifuged at 10,500× *g* (Sartorius AG, Gottingen, Germany) and 4 °C for 30 min. The proteolytic activity of the supernatant was immediately determined as described in Section 3.5.

### 3.3. Effect of pH on Extraction of BGP

To test the effect of extraction pH, a series of buffers was used in the standard extraction procedure described above. Ginger rhizome (100 g) was washed, cut into fine pieces and homogenized separately with 100 mM potassium phosphate buffer (200 mL, pH 6.4, 6.8, 7.0 and 7.2) and 100 mM Tris-HCl buffer (200 mL, pH 7.6, 8.0, 8.4, and 8.8). The resulting homogenates were filtered through a piece of cheesecloth and the filtrates were centrifuged at 10,500× *g* and 4 °C for 30 min. The supernatants were then assayed for proteolytic activity. The optimum extraction pH was chosen based on its performance on producing BGP with highest relative protease activity.

### 3.4. Effect of Stabilizers on Extraction of Ginger Protease

To test the effect of stabilizers, three additives were examined for their ability to protect the activity of ginger protease during extraction. Based on the standard extraction procedure, ginger rhizomes (100 g) were washed, cut into fine pieces and homogenized with 100 mM potassium phosphate buffer (200 mL, pH 7.0) containing either of the following stabilizers: 0.2% ascorbic acid, 0.2% ascorbic acid and 5 mM EDTA, or 10 mM cysteine and 5 mM EDTA. After filtration and centrifugation, the proteolytic activity of the supernatant was measured and compared with control (without any stabilizers) to determine the most effective stabilizer. The control was extract (supernatant) without the addition of any stabilizer.

### 3.5. Proteolytic Activity Assay

A colorimetric method adopted from Adulyatham and Owusu-Apenten [8] was used to measure the activity of BGP with a slight modification. The substrate azocasein (1 mg/mL) was prepared in 100 mM potassium phosphate buffer (pH 7) prior to the analysis. One milliliter of azocasein solution was pipetted into a 2 mL Eppendorf tube containing buffer solution (0.6 mL) and enzyme extract (supernatant, 0.1 mL) to initiate the reaction. After mixing, the reaction mixture was incubated for 20 min at 60 °C. The reaction was terminated with the addition of trichloroacetic acid (TCA, 0.3 mL, 10% w/v) solution, followed by centrifugation at 9,000× *g* at room temperature for 10 min (Sartorius Model 3–18 k, Sartorius AG, Weender Land Strasse, Gottingen, Germany). The absorbance of the supernatant was then measured at 410 nm. A blank was prepared as above except that the enzyme was first heated in a boiling water bath for 5 min. One unit (U) of protease activity was defined as the amount of the enzyme that cause an increase in absorbance by 1 absorbance unit per mL extract per minute. Specific activity is expressed as U/mg protein.

### 3.6. Protein Concentration Determination

Protein content of the crude extract and dried BGP was determined using Lowry method [27] using bovine serum albumin (Sigma Chemical Co., St. Louis, MO, USA) as standard. The protein content was expressed as mg/mL for crude extract.

### 3.7. Production of BGP Powder

To produce dried BGP, a total of 100 g of ginger rhizome was used. The enzyme was first extracted from the rhizome as described above using 100 mM potassium phosphate buffer (200 mL, pH 7.0) containing 10 mM cysteine and 5 mM EDTA. After centrifugation at 10,500× *g* (Sartorius Model 3–18 k, Sartorius AG) and 4 °C for 30 min, the supernatant was filtered through Celite (diatomaceous earth) to remove any suspended materials and then mixed with 60% ammonium sulfate to partially concentrate and purify the enzyme. The precipitate that was obtained was collected by centrifugation at 10,500× *g* and 4 °C for 30 min and then resolubilized in a minimal volume of 50 mM potassium phosphate buffer (pH 7.0). The enzyme solution was dialyzed against two changes of 50 mM potassium phosphate buffer (pH 7.0) containing 1 mM EDTA for 16 h at 4 °C. The dialysate was centrifuged at 10,500× *g* and 4 °C for 20 min to remove any insoluble substances and then lyophilized using a freeze dryer (Model-7753032 Freeze Dry System, Labconco, Kansas City, MO, USA). The yield of dried BGP was determined gravimetrically while the proteolytic activity was measured as described above using a 1.0 mg dried BGP/mL solution. The BGP was then stored at −20 °C prior to further analysis. The production of BGP powder was conducted in triplicate.

### 3.8. Characterization of Bentong Ginger Protease

#### 3.8.1. Determination of Protease Type

Four different protease inhibitors were used to classify the type of Bentong ginger protease under study. Ten millimolar solutions of *o*-phenanthroline, phenylmethylsulfonyl fluoride (PMSF), EDTA and *N*-ethylmaleimide (NEM) in 100 mM potassium phosphate buffer (pH 7.0) were prepared prior to the analysis. The activity of 1.0 mL of BGP solution (1.0 mg/mL dried BGP) pre-incubated with 1.0 mL of each inhibitor solution for 1 h at 25 °C was determined using the standard proteolytic method as described above. The activity obtained is expressed as relative activity based on the percentage of proteolytic activity of inhibitor-treated enzyme against the control (without added inhibitor). The protease type of BGP was determined based on the degree of inhibitory effect.

#### 3.8.2. Optimum Temperature

Determination of the optimum temperature of BGP was conducted by assaying the enzyme activity at various temperatures. The enzyme solution (1.0 mg/mL dried BGP solubilized in 100 mM potassium phosphate buffer, pH 7.0) was prepared prior to analysis. Its activity was then assayed at temperatures ranging from 20 to 90 °C using 1 mg/mL azocasein as the substrate for 20 min using the proteolytic assay as described above.

#### 3.8.3. Optimum pH

The optimum pH of BGP was determined by measuring the activity of the enzyme at different pH values ranging from pH 3 to 10. The buffers used were as follows: 100 mM citrate buffer (pH 3.0–5.0), 100 mM potassium phosphate buffer (pH 6.0 and 7.0), 100 mM Tris-HCl buffer (pH 8.0 and 9.0) and 100 mM sodium carbonate buffer (pH 10). Azocasein was dissolved in the respective buffers to obtain 1 mg/mL solutions and then used to assay the activity of a solution of 1 mg/mL of dried BGP. The method of proteolytic assay was as described above.

#### 3.8.4. Effect of Cations and Detergents

The effect of cations on activity of BGP was determined by including 0.1 mL of 10 mM cation (K^+^, Na^+^, Li^+^, Mg^2+^, Ca^2+^, Ba^2+^, Co^2+^, Zn^2+^, Cu^2+^, Hg^+^ and Al^3+^) solutions which were prepared from their chloride salts in separate proteolytic assay reaction mixtures. BGP solution containing 1.0 mg/mL of dried BGP was prepared prior to the proteolytic activity analysis as described above. The effect of detergents in the activity of BGP was tested by adding 0.1 mL of 5 mM (to give a final concentration of 0.25 mM) of EDTA, Triton X-100, sodium dodecyl sulfate (SDS), Tween 20 or Tween 80 into separate reaction mixture. The activity obtained is expressed as relative activity based on the percentage of proteolytic activity of treated enzyme against the control (without added cation/detergent).

#### 3.8.5. Determination of K_m_ and V_max_

The *K_m_* and V_max_ BGP were determined using azocasein as a substrate at selected optimum temperatures for 10 min based on the assay method described above. One mg/mL of BGP was prepared prior to the analysis and the concentrations of substrate used in this study were 0.1, 0.5, 1, 1.5 and 2.0 mg/mL. The *K_m_* and V_max_ were calculated as described by Lineweaver-Burk [28].

#### 3.8.6. Storage Stability

To determine the storage stability, BGP (1 mg) was placed in an Eppendorf and stored at −20 °C; 4 ± 1 °C and room temperature for up to 90 days. The BGP was then dissolved in 100 mM potassium phosphate buffer (1 mL) prior to the analysis. The activity obtained was expressed as relative activity as described above. The half-life (t_1/2_) of the BGP was calculated by fitting the data of natural log (ln) activity *versus* time (day) [8].

### 3.9. Statistical Analysis

The experiments were conducted in triplicate. The data which were presented as mean ± standard deviation was analyzed using Analysis of Variance (ANOVA) with Minitab 14 (State College, PA USA, 2003) statistical software.

## 4. Conclusions

The protease extracted from ginger rhizome var. Bentong by pH 7 phosphate was classified as a cysteine protease showing optimum activity at high temperature of 60 °C and over a wide range of pH values from 6 to 8. The enzyme could react with substrate with a higher rate of hydrolysis and demonstrated high storage stability at low temperatures. The properties of BGP obtained in this study should facilitate its application in the food and other industries.
